# Interplay Between Diabetes and Pancreatic Ductal Adenocarcinoma and Insulinoma: The Role of Aging, Genetic Factors, and Obesity

**DOI:** 10.3389/fendo.2020.563267

**Published:** 2020-09-30

**Authors:** Bertrand Duvillié, Rayane Kourdoughli, Sabine Druillennec, Alain Eychène, Celio Pouponnot

**Affiliations:** ^1^Department of Signaling, Radiobiology and Cancer, Institut Curie, Orsay, France; ^2^INSERM U1021, Centre Universitaire, Orsay, France; ^3^CNRS UMR 3347, Centre Universitaire, Orsay, France; ^4^Université Paris-Saclay, Orsay, France; ^5^PSL Research University, Paris, France

**Keywords:** diabetes, pancreas, cancer, aging, insulinoma, obesity

## Abstract

Epidemiologic analyses have shed light on an association between type 2 diabetes (T2D) and pancreatic ductal adenocarcinoma (PDAC). Recent data also suggest a potential relationship between T2D and insulinoma. Under rare circumstances, type 1 diabetes (T1D) can also be implicated in tumorigenesis. The biological mechanisms underlying such relationships are extremely complex. Some genetic factors contributing to the development of T2D are shared with pancreatic exocrine and endocrine tumors. Obesity and overweight can also contribute to the initiation and severity of T2D, while aging may influence both endocrine and exocrine tumors. Finally, pharmacological treatments of T2D may have an impact on PDAC. On the other hand, some treatments for insulinoma can trigger diabetes. In the present minireview, we discuss the cellular and molecular mechanisms that could explain these interactions. This analysis may help to define new potential therapeutic strategies.

## Introduction

Diabetes is a metabolic disorder characterized by chronic hyperglycemia. Type 1 diabetes (T1D) is less frequent (5.6%) than type 2 diabetes (T2D) and is caused by autoimmune destruction of pancreatic beta-cells. T2D represents 91.2% of diabetes cases and is generally associated with insulin resistance and compensatory hyperinsulinemia, an early indicator of metabolic dysfunction. In the longer term, T2D leads to progressive functional defects of beta-cells. The remaining cases are primarily gestational diabetes, which are represented by hyperglycemia that generally disappears after delivery. Pancreatic adenocarcinoma (PDAC), the most frequent (95%) exocrine pancreatic cancer, is also the most lethal, with a 5-year overall survival of less than 8% (1). Insulinomas are functional neuroendocrine tumors originating from beta-cells. They are generally benign but can metastasize in 5%–10% of cases (2). Interestingly, T2D and pancreatic cancers share several common risk factors, and long standing T2D represents a recognized risk for carcinogenesis. Inversely, PDAC may also be responsible for diabetes (3). This link between diabetes and cancer was first suggested by epidemiologic observations. In particular, PDAC is strongly associated with diabetes (4). Although T2D is a well-established risk factor for PDAC (5), this association is less clear for T1D. Indeed, a clinical prospective study on patients with T1D showed an increased risk of stomach, cervical and endometrium cancers, but only a very modest association with PDAC (6). These differences between T1D and T2D in the risk of developing PDAC may be attributed to differences in insulin levels, which is a risk factor (6). Finally, recent data has suggested a potential association between T2D and insulinoma (7). Despite this evidence, the causative link between T2D and PDAC, as well as insulinoma is not completely understood. The possible molecular mechanisms of this association will be discussed in this review.

## Common Determinants of Diabetes and Pancreatic Cancer

**Figure 1:**
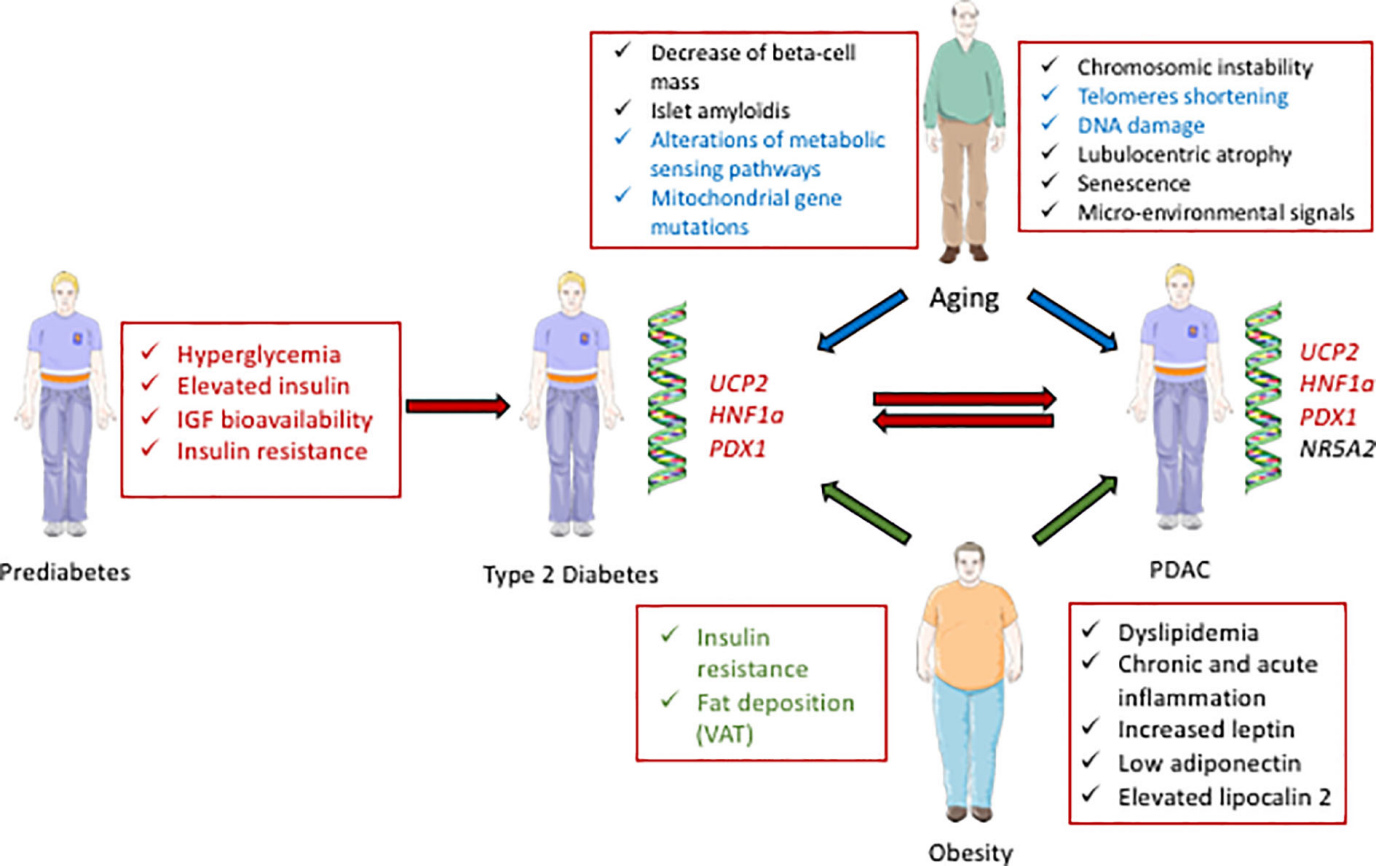
Schematic representation of the interactions between diabetes and PDAC. Some biological parameters occurring during prediabetes, including hyperglycemia, elevated insulin, and IGF bioavailability contribute to diabetes (T2D) and can further lead to PDAC (red arrows). Some genetic factors; *UCP2*, *HNF1a*, and *PDX1*, are also common determinants of diabetes and PDAC (written in red). Some parameters in relation with aging can cause T2D and/or PDAC (blue arrows, and the common determinants for T2D and PDAC development are written in blue). Some characteristics of obesity can contribute to T2D and/or PDAC (green arrows, and the common determinants for T2D and PDAC development are written in green).

T2D and PDAC have common determinants, including aging, obesity, and genetic factors (**Figure 1**), in addition to some environmental factors that include tobacco smocking, alcohol consumption and low level of physical activity (8). T2D and insulinoma also share causative signals.

### Aging

#### The Effect of Aging in T2D

Aging is the time-dependent deterioration of physiological functions affecting both diabetes and PDAC. The incidence of diabetes increases with age: with 1.8 million patients aged 20–39, 11.7 millions at 40–59, to 19.3 millions at 60–79 in the European Union in 2017 (9). Moreover, the mass of islet cells increases during maturation, but slowly decreases after age 40 (10). Islet amyloidis, which is associated with insulin resistance and T2D, is more commonly found in older individuals (11). Finally, metabolic sensing pathways, such as the mTOR, AMP-activated protein kinase, and insulin/insulin-like growth factors (IGFs) pathways (12, 13) are age-dependent. Interestingly, mTOR, a kinase activated by metabolic signaling, also plays an important role in T2D (14) and PDAC (15).

#### Aging and PDAC

Aging dramatically increases the risk of pancreatic carcinogenesis (16). Indeed, pancreatic intraepithelial neoplasia (PanINs) is one precursor of PDAC. In an autopsy study, high grade PanIN lesions were found more frequently in T2D patients and old individuals, suggesting a role of both aging and diabetes (17). Moreover, PanINs are associated with chromosomal instability, telomere shortnening (18), and DNA damage, which all depend on aging (19). Several cellular mechanisms involved in aging also play an important role in PDAC. For example, lobulocentric atrophy, a combination of atrophy of acinar parenchyma, acinar to ductal metaplasia and fibrosis, promotes proliferation of small ductular structures and PanINs. This process is found in patients at high risk of PDAC and is age-dependent (20). Moreover, senescence is also important in cancer cells: after oncogenic transformation, cells can undergo senescence, with a reduction of their proliferation. However, some malignant cells often escape this process (21). In addition, age-related senescent-associated secretory phenotype (SASP) cells in the stromal micro-environment support cancer progression (21). In the KRAS^G12D^ model of PDAC, knock-out of the senescence-inducing factor SIN3B reduced the initiation and progression of pancreatic lesions, while decreasing secretion of the SASP factor IL-1α (22). Moreover, the conditional knock-out of IL-1α also reduces the number of neoplastic lesions. Finally, mitochondrial gene mutations, which accumulate with age, affect cell metabolism. Consequently, a selective growth advantage that promotes cancer is confered to the cells present in the aging environment (23, 24). Importantly, such mitochondrial events also enhance tumor progression in PDAC (25). Together, these data highlight the strong impact of aging in PDAC.

#### The Role of Aging in Insulinoma

*Men1* knock-out mice provide a model of insulinoma, in which tumors develop late. Indeed, early inactivation of *Men1* specific to beta-cells leads to multiple insulinoma only by 60 weeks (26), suggesting the requirement of additional somatic events. Notably, in *Men1* knock-out tumors, an increase in the number of entire chromosome 11 was also found in insulinomas, and of chromosome 15 in pituitary prolactinomas. Several oncogenes, including *c-MYC* and *ErbB2/Her2/Neu* are present in these duplicated regions (26). The age-related penetrance of MEN1 in patients is 7%, 52%, 87%, 98%, 99%, and 100% at 10, 20, 30, 40, 50, and 60 years of age, respectively (27), suggesting that aging also influences tumorigenesis in human MEN1 tumors. Interestingly, different phenotypes in MEN1 monozygotic twins were observed: in (28), both twins developed parathyroidism, but only one had a pancreatic tumor. This observation suggests that one single mutation in *MEN1* is insufficient to induce insulinoma. The variant *V109G* of p27 and inactivating mutations of *CDKN1B* were shown to influence the clinical phenotype of MEN1 patients (29). Moreover, some cell-cycle regulators, whose expression is age-dependent (30), are also differentially expressed in human beta-cells from insulinoma as compared to healthy tissue (31). For example, p16 is more heavily expressed at the adult stage than in prenatal beta-cells (32). Such expression restricts beta-cell proliferation with aging (30) and also promotes senescence. Interestingly, its expression is considerably reduced in insulinoma cells (31). Together, these data suggest that aging influences beta-cell proliferation, but that insulinoma cells develop a specific proliferation pattern evading this control.

### Obesity

#### Obesity and Diabetes

Obesity, characterized by excessive accumulation of body fat, with a body mass index (BMI) of 30 kg/m^2^ or greater, is a well-known risk factor of diabetes (**Figure 1**) (33). Indeed, 87.5% of adults with T2D are also obese or overweight [BMI>25 (34, 35). The first causative link between obesity and T2D is insulin resistance. Indeed, both obesity and insulin resistance precede altered glycemia (36). Moreover, fat deposition has deleterious effects that depend on its anatomical location. The visceral adipose tissue (VAT), located in the abdominal cavity is linked to a higher risk of T2D as compared to subcutaneous adipose tissue (SCAT) (37).

#### Obesity and PDAC

Infiltration of adipose tissue favors pancreatic precancerous lesions (38). Indeed, in obese patients, fat has an effect on PanIN lesions and PDAC development. Obesity promotes inflammation that activates tumor associated neutrophils, and consequently pancreatic stellate cells, leading to increased desmoplasia and tumor growth (39). Moreover, chronic inflammation can promote EMT in PanIN cells, driving tumor progression and cell dissemination, leading to PDAC (40). Insulin resistance associated with obesity promotes dislypidemia (41), with elevated concentrations of triglycerides (42), and increased cholesterol synthesis (43). Hypertriglyceridemia is the third most common cause of acute pancreatitis (44), which also represents a risk for PDAC (45). Indeed, patients with acute pancreatitis had a 2-fold increased risk of pancreatic cancer when compared to the matched population. Moreover, adipokines, which include leptin, adiponectin, and lipocalin 2, also establish a connection between obesity and PDAC. In animals and humans, the leptin receptor Ob-Rb plays an important role in obesity (46). While leptin is produced by mature adipocytes, human PDAC cell lines and tissues both express the leptin receptor. Overexpression of leptin in an orthotopic model of human pancreatic cancer promotes tumor growth and lymph node metastasis (47), indicating that leptin is a key factor for PDAC. Recently, a possible interconnection between leptin and the Notch pathway, which is responsible for transformation, proliferation, tumor progression, EMT and chemoresistance, was described (48). Adiponectin is found in adipose tissue and its expression is very low in obese subjects. Prospective epidemiologic studies have shown that low concentration of adiponectin is linked to a higher risk of PDAC (49). Interestingly, adiponectin treatment inhibits the proliferation of human pancreatic cancer cells (50). Knocking-down adiponectin receptors abolished these effects, and enhanced the growth of human pancreatic cancer xenografts in nude mice. Moreover, this antiproliferative effect of adiponectin was shown to be mediated by the β-catenin signaling pathway. However, the roles of leptin and adiponectin are still debated, as a higher adiponectin/leptin ratio and lower leptin levels were found in patients with PDAC as compared to controls (51). Finally, lipocalin 2, a protein involved in innate immunity, also plays an important role in the cellular microenvironment that contributes to PDAC. Lipocalin 2 was found to be a regulator of VAT hypertrophy in animals treated with high fat diet (HFD). The deletion of *Lipocalin2* decreases PDAC incidence in KRAS-G12D transgenic mice (52). More generally, diet has a strong impact on pancreatic cancer. Recently, Chang et al. showed that HFD dramatically increases the incidence of PDAC in KRAS-G12D mice. Indeed, the PanIN lesions express new genetic variants, suggesting that genetic alterations may participate to this process (53).

#### Obesity and Insulinoma

Obesity has thus far not been identified as a cause of insulinoma, but the reverse has been described. Insulinoma can be linked to hyperphagia in some cases by induction of hypoglycemia and hunger. This may lead to weight gain in 20%–40% of patients and even to overt obesity (54, 55). Interestingly, the orexigenic hormone ghrelin is associated with obesity (56). Co-expression of ghrelin and its receptor was detected in several pancreatic endocrine tumors, and specifically in insulinoma, but elevated circulating ghrelin is rare in these patients (57).

### The Genetic Factors

#### The Genetics of Diabetes and PDAC

Genome wide association studies (GWAS) have been used to identify relationships between diabetes and pancreatic cancers. Several pancreatic developmental genes, *NR5A2*, *PDX1*, and *HNF1A*, were identified as susceptibility factors for PDAC (**Figure 1**) (58). Moreover, heterozygous mutations in some of these genes, *PDX1* and *HNF1α*, are also responsible for different monogenic forms of maturity onset diabetes of the young (MODY 4 and MODY 5). Some variants of *PDX1* and *HNF1α* are also associated with increased risks of T2D (59, 60), obesity (61), or hyperglycemia (62). The antioxidant mitochondrial uncoupling protein 2 (UCP2), which controls pancreatic development and insulin secretion (63), is overexpressed in PDAC tumors compared to normal adjacent tissues (64), suggesting that *UCP2* overexpression is a biomarker of bad prognosis. However, other recent studies using the pancreatic cancer cell line Mia PACA2 showed that UCP2 inhibits cancer cell proliferation and tumorigenesis (65). This effect is mediated by retrograde mitochondrial signaling on the Warburg effect that reorients mitochondrial function toward oxidative phosphorylation rather than glycolysis. Additional analyses are needed to elucidate the discrepancy between these two studies involving *UCP2*. Taken together, these data indicate a link between genes controlling pancreas development, diabetes and PDAC.

#### The Genetic Links Between Diabetes and Insulinoma

**Figure 2:**
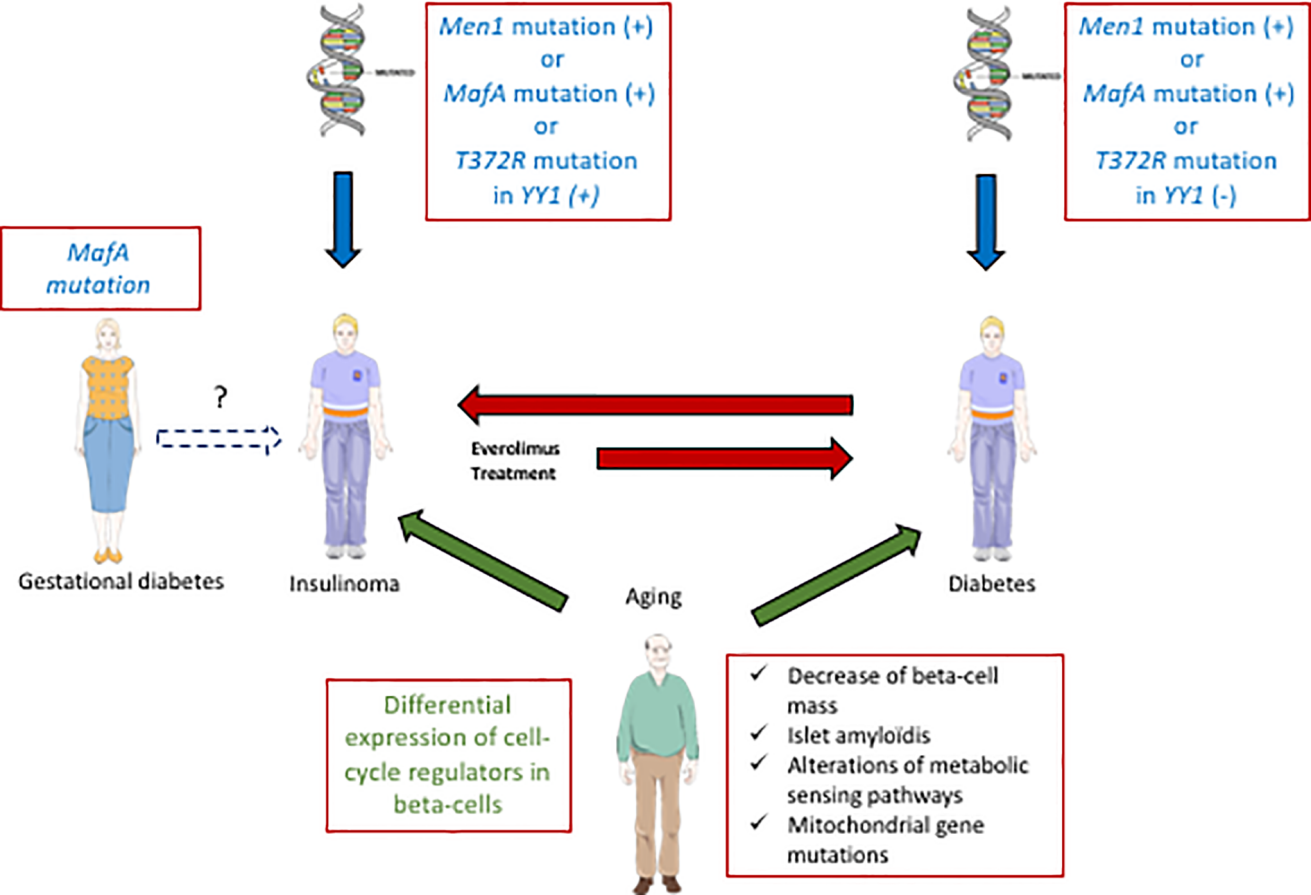
Interactions between diabetes and insulinoma. Some specific *MafA* mutations can predispose to diabetes (T2D) or insulinoma. In some rare cases with such mutations of *MafA*, gestational diabetes precedes insulinoma. Moreover, other genes (*Men1*, *YY1*) are also involved positively (+) or negatively (−) in the development of diabetes or insulinoma (blue arrows). Aging also contributes to both pathologies (green arrows, common aging determinant for T2D and insulinoma written in green). Some inulinoma treatments (everolimus) can enhance the risk of diabetes (red arrow). On the other hand, exceptional cases of T1D can induce insulinoma (red arrow).

Recently, a strong link between T2D and insulinoma has been established (**Figure 2**). A p.Ser64Phe mutation in *MAFA* that prevents GSK3-mediated MAFA phosphorylation (42, 44) was identified in 25 individuals from two independent families (7). These patients develop either insulinoma or diabetes with 90% penetrance. Interestingly, the MAF proteins are well established oncoproteins (66) and their tumorigenic activity is regulated by GSK3-mediated phosphorylation in different cancers (67, 68). Moreover, other studies have also revealed a role of *MAFA* in diabetes. Within the pancreas, *MAFA* is exclusively expressed in developing and mature beta-cells. MafA activates the insulin promoter in response to glucose, and regulates genes involved in beta-cell function such as glucose transporter 2, glucagon-like peptide 1 receptor and prohormone convertase 1/3 (69). Accordingly, glucose-stimulated insulin secretion (GSIS) is impaired in *MafA* knock-out mice, and the architecture of the islets is disorganized. Moreover, these mice develop T2D at 50 weeks after birth (70). In humans, data have also established a link between *MAFA* and different forms of diabetes. Indeed, expression of *MAFA* is decreased in islets from T2D patients and a polymorphism in *MAFA* is associated with T1D (71).

Cases of insulinoma with pre-existing diabetes are very rare (72, 73). In (7), a 27 year-old female patient with a *MAFA* mutation preventing its phosphorylation by GSK3 first developed gestational diabetes, and subsequent insulinoma at 55 years old. This finding suggests that the *MAFA* mutation may have caused metabolic disorders in relation to diabetes, that would lead to insulinoma in the long term. Another genetic link between diabetes and insulinoma was suggested by the presence of a recurrent somatic T372R mutation in YY1 (Yin and Yang 1 protein) in 30% of tumors (74). YY1 is a transcription factor that belongs to the GLI-Kruppel class of zinc finger proteins and is a target of mTOR inhibitors. In beta-cells, YY1 regulates the transcription of CXCL12, which also has antidiabetogenic potential (75). In some cases, insulinoma develop in a context of hereditary predisposition to pancreatic endocrine tumors. Indeed, *MEN1*, type-1 multiple endocrine neoplasm, represents the most frequent predisposition gene for insulinoma (76). Moreover, a relationship between *MEN 1* and *MAFA* genes was also established: altered *MEN1* expression was shown to disrupt the *MAFA* differentiation pathway in human and mice insulinoma cells (77).

## Is Diabetes a Risk Factor for Pancreatic Cancers?

### Hyperglycemia

#### Hyperglycemia and PDAC

In T2D, hyperglycemia is caused by excessive hepatic gluconeogenesis, decreased incretin activity, and peripheral glucose uptake, as well as altered insulin signaling. T2D results from a long history of metabolic disorders before diagnosis. These events can cause carcinogenesis and particularly PDAC (**Figure 1**) (76). Indeed, patients can remain asymptomatic for many years, with undiscovered glucose intolerance and transient hyperglycemia (78). This prediabetic period considerably increases the probability of developing PDAC (79). One possible mechanism is the activation of the TGFβ pathway by glucose, leading to reduced E-cadherin levels in pancreatic ductal cells and to a pronounced mesenchymal phenotype promoting tumor growth and metastasis (5). Hyperglycemia may also increase genomic instability leading to *KRAS* mutations through activation of O-GlcNAcylation and nucleotides deficiency (80). Finally, the mTOR pathway controls protein synthesis and autophagy, and its deregulation is implicated in diabetes, cancer, and the aging process (81). Interestingly, mTOR inhibition in mouse models of KRAS-dependent PDAC subtypes reduces tumorigenesis (15).

#### Hyperglycemia and Insulinoma

Glucose controls beta-cell proliferation both *in vitro* and *in vivo* (82). In the context of insulin demand, beta-cells undergo hypertrophy or hyperplasia to normalize glycemia (83). One hypothesis is that pre-existing diabetes leads to insulinoma through hyperglycemia. However, such cases are extremely rare. Recently, two patients with pre-existing T1D developed insulinoma (84, 85). In (84), a 31-year-old man experienced T1D for 28 years. Surprisingly, frequent hypoglycemic episodes occurred, leading to the arrest of insulin therapy. After resection, histopathology revealed a grade-2 insulinoma. One unsolved issue is the absence of an autoimmune response against tumor cells in this patient (84). Further analyses will be necessary to investigate the mechanisms involved in tumor progression in such patients.

### Insulin and Insulin-Like Growth Factors (IGFs) in Diabetes and Tumorigenesis

The insulin/IGF signalization plays an important role in diabetes. Epidemiological studies have associated serum level variations of IGF-1 (86, 87), IGF-2 (88), IGF binding proteins 1 (89, 90), 2 (91), 3 (86, 90), 4 (91) with T2D. Moreover, obese subjects also exhibit alterations of the IGF system (88, 92, 93) influenced by the presence or absence of T2D (93). Non-diabetic obese subjects have elevated free IGF-1 and IGF-2, total IGF-2, IGF-BP3, and reduced IGF-BP1 and 2 levels. In obese T2D patients, IGFBP-2 is further reduced (93).

#### PDAC

PDAC originates from both ductal and acinar cells of the pancreas (94), which are exposed to high levels of insulin. Such proxicrine signals promote growth of pancreatic cancer cells (**Figure 1**) (3). Indeed, the effects of insulin and IGFs 1 and 2 are mediated by the insulin receptor (IR) and IGF1 receptor (IGF1R) (95, 96). As previously discussed, obesity and T2D are associated with increased risk of PDAC. These metabolic disorders are characterized by insulin resistance, compensatory overproduction of insulin and increased bioavailability of IGF-1 (97). To examine the role of insulin in PDAC initiation, Ptf1aCre^ER^LSL-KRAS^G12D^*Ins*1^+/−^*Ins*2^−/−^ mice, which have a sustain reduction of insulin but no altered glycemia, were used (98). Mice with reduced insulin had a significant decrease in the number of PanINs and pancreatic tumors when compared to controls. Thus, these results demonstrate that insulin regulates PDAC development. Interestingly, altered expression of the tumor suppressor p53, observed in 50% to 75% of PDAC (99), was shown to stimulate the insulin/IGF1 pathway (100). Moreover, polymorphisms in the IGF genes have been associated with decreased survival of patients with PDAC (101). Taken together, these data strongly support a role of the insulin/IGF axis in pancreatic cancer.

#### Insulinoma

In animals and human, some associations between IGF2 and diabetes have been shown. In particular, IGF2 overexpression in transgenic mice leads to beta-cell dysfunction (102), by inducing beta-cell de-differentiation and reticulum stress. Moreover, in a mouse model of multistage carcinogenesis induced by the SV40 large T antigen in pancreatic beta-cells, IGF2 was increased and contributed to insulinoma development (103). Recently, studies have shown that the IGF pathway is activated in insulinoma (104). Glutamine can also stimulate biosynthesis and secretion of IGF2 in mouse insulinoma cells, which regulate beta-cell mass and function in an autocrine manner (105). Interestingly, hypermethylation of the differentially methylated region 2 of *IGF2* was discovered in human insulinoma, leading to loss of imprinting and overexpression of *IGF2* (106). Finally, IGF2 overexpression was also detected in *Men1-*mutated mouse insulinoma (107). IGF signaling thus appear to be an important hallmark of insulinoma.

## Diabetes Treatment to Prevent Pancreatic Cancers?

### PDAC

Metformin (108) is the most frequently prescribed, first-line treatment drug for T2D (109). Metformin decreases glycemia by lowering hepatic gluconeogenesis and improves insulin sensitivity by promoting glucose uptake in skeletal muscle and adipose tissue. Several epidemiological studies have demonstrated that metformin administration reduces incidence, recurrence and mortality of pancreatic cancer in diabetic patients (110, 111). Clinical trials using metformin in combination with other drugs used to treat PDAC are actually under investigation (https://clinicaltrials.gov/ct2/results?term=metformin&cond=Pancreatic+Cancer). To delineate the molecular mechanisms involved in this protective effect of metformin, animal models were used. Metformin significantly decreased the incidence of PDAC promoted by diet-induced obesity in the conditional KRAS-G12D knock-in mouse (112). Together, these findings demonstrate that metformin, a treatment for diabetes, represents an important pharmacological tool for PDAC prevention, strengthening the link between these two pathologies. Moreover, AdipoRon, which acts as an adiponectin receptor agonist (113), has antidiabetic properties and can inhibit tumor growth of pancreatic cancer cells MIAPACa-2 in xenografts. AdipoRon can also induce cell death in cells derived from PDAC patients (114). These data suggest that AdipoRon could be a therapeutic agent for both diabetes and PDAC. Finally, several studies analyzed the antidiabetic and protective effects of aspirin against PDAC. Indeed, inflammation is a hallmark of T2D, and aspirin reduces inflammation by regulating T-cell function (115). Interestingly, clinical analysis of a subgroup of patients with diabetes showed a protective role of aspirin against PDAC (116). However, the effects of aspirin against PDAC seem to be heterogenous and controversial (116–118). Further analysis is thus required to better understand these effects.

### Insulinoma

Anti-cancer drugs are used to treat insulinoma. Recently, a combination of mTOR inhibitors and streptozotocin was shown to have synergistic antitumor effects in insulinoma cells, both *in vitro* and *in vivo* (119). Everolimus, an mTOR inhibitor, was successfully used to treat advanced pancreatic neuroendocrine tumors in a phase 3 clinical trial (120) (RADIANT-3 ClinicalTrials.gov number, NCT00510068). However, other data indicate that such anti-cancer therapy also has endocrine side effects, such as increased plasma triglycerides, LDL cholesterol, and high incidence of hyperglycemia (121). Thus, despite its benefits in cancer, this treatment may enhance the risk of diabetes.

## Conclusion

Pre-clinical and clinical data provide clear evidence of common characteristics shared by T2D and PDAC, as well as T2D and insulinoma. The association between diabetes and PDAC is frequent, while it is more unusual between diabetes and insulinoma. Some specific gene mutations contribute to both T2D and insulinoma, strengthening the link between these diseases, while others mutations have opposite effects on T2D and insulinoma. Diabetes and PDAC share several metabolical disorders, that are also found during obesity. Accordingly, obesity often contributes to PDAC initiation, whereas obesity is a consequence of insulinoma. Understanding the relation between T2D and PDAC and between T2D and insulinoma may have important consequences. Indeed, treatments of T2D can limit PDAC progression, while treatment for insulinoma may induce T2D. These important findings should be taken into consideration to develop new pharmacological strategies to limit tumor progression.

## Author Contributions

All authors contributed to the article and approved the submitted version.

## Funding

BD is supported by Société Francophone du Diabète (SFD) (Grant number 26866).

## Conflict of Interest

The authors declare that the research was conducted in the absence of any commercial or financial relationships that could be construed as a potential conflict of interest.
